# Bioactive Compounds Intake of the Brazilian Population According to Geographic Region

**DOI:** 10.3390/plants12132414

**Published:** 2023-06-22

**Authors:** Renata A. Carnauba, Flavia M. Sarti, Neuza M. A. Hassimotto, Franco M. Lajolo

**Affiliations:** 1 Department of Food Science and Experimental Nutrition, School of Pharmaceutical Sciences, University of São Paulo, São Paulo 05508-220, Brazil; aymoto@usp.br (N.M.A.H.); fmlajolo@usp.br (F.M.L.); 2 Food Research Center, CEPID-FAPESP (Research Innovation and Dissemination Centers, São Paulo Research Foundation), São Paulo 05508-020, Brazil; 3 Center for Research in Complex Systems Modeling, School of Arts, Sciences and Humanities, University of São Paulo, São Paulo 05508-220, Brazil; flamori@usp.br

**Keywords:** plant biodiversity, food plants, bioactive compounds, polyphenols, carotenoids, phytochemicals

## Abstract

Studies have been conducted in order to estimate bioactive compound consumption across populations, with substantial disparities according to the origin of the cohort examined. In this sense, Brazil is a continental country with marked differences in food plant availability across geographic regions. We aimed to estimate the bioactive compound intake according to Brazilian geographic region, as well as to determine the major contributors. Data were obtained from the National Dietary Survey 2017–2018, a cross-sectional population-based study including data on the individual food intake of 46,164 subjects aged ≥10 years. The consumption of polyphenols (total and classes) was significantly higher in the South compared with other regions (*p* = 0.0001). Total carotenoid intake was higher in the Midwest, followed by the Southeast (*p* = 0.0001). Tea was the main supplier of total polyphenol intake in the South, whereas coffee contributed the most to total polyphenol intake in other Brazilian regions. Açaí, caja juice, mango and corn were important suppliers of carotenoid intake in the North and Northeast. Bioactive compound intake presented variations according to Brazilian region, and individuals living in the South, Midwest and Southeast may experience higher bioactive-dense diets. We highlight the potential of many food plants for sustained explorations to the development of marketable products, possibly increasing the bioactive compound intake.

## 1. Introduction

Brazil is a country of continental dimensions with marked differences in food availability according to geographical region [1,2]. The vast territory and favorable climatic conditions throughout the six different Brazilian biomes (Amazon, Caatinga, Cerrado, Atlantic Forest, Pampas and Pantanal) play a major role in the great diversity observed in plant species [3,4]. The North of Brazil presents a great variety of native fruits, such as açaí, buriti and camu-camu [5]. The Northeast region had a high availability of cereals and tuberous roots, mainly corn and cassava [6], whereas the Midwest presents fructiferous native species such as pequi, buriti and baru [3]. Finally, the Southeast and South regions had a great availability of cold climate plant species, such as araçá, jaboticaba, uvaia and cerejeira do Rio Grande [7].

The mentioned difference in plant biodiversity according to Brazilian geographical region may also impact diet quality [8,9], including bioactive compound intake [4,10]. Bioactive compounds are a large and heterogeneous group of metabolites present in diverse plant-based foods, such as fruits, vegetables, cereals, beans and plant-based beverages. According to their chemical structure, bioactive compounds can be divided into several classes and subclasses, polyphenols and carotenoids being the most studied classes of food bioactive compounds [11]. Accumulating evidence indicates that the consumption of bioactive-dense foods may play a role in reducing the risk of a wide range of diseases linked to oxidative stress and inflammation, such as cardiovascular diseases [12,13,14], type 2 diabetes [15,16], neurodegenerative diseases [17,18,19] and cancer [20,21,22,23,24].

Considering the health impact of bioactive compound intake, a growing body of research has been conducted in order to estimate its consumption across populations [25,26,27,28,29,30,31,32,33,34,35]. The studies report substantial differences according to the origin of the cohort examined, which may be related to the different food availability, cultural background and individual preferences. In addition, the majority of studies are developed in European countries [26,27,31,32,33,34], and information on Latin American countries is still scarce [25,35]. In Brazil, a previous study estimated total and individual polyphenol intake in a representative sample of the Brazilian population using data from the National Dietary Survey (NDS) 2008–2009 and 2017–2018, showing wide variation in consumption according to Brazilian geographic regions [36].

Thus, the purpose of the present study was to describe the polyphenol (total, classes and subclasses) and carotenoid (α-carotene, β-carotene, β-cryptoxanthin, lycopene, lutein, neoxanthin, violaxanthin and zeaxanthin) intake according to the five Brazilian geographic regions (North, Northeast, Midwest, Southeast and South) using data from the NDS 2017–2018, as well as to determine the major food contributors to the intake in each region.

## 2. Results

Information on 46,164 participants was available for analysis, comprising 6836 individuals from the North region, 16,097 from the Northeast, 5740 from the Midwest, 11,471 from the Southeast and 6020 from the South (Table 1).

Total classes and subclasses of polyphenol energy-adjusted intake across the Brazilian region are presented in Table 2. The consumption of total polyphenols, phenolic acids, flavonoids and other polyphenols was significantly higher in the South (*p* = 0.0001) compared with other regions. The second region with higher intakes of total polyphenols and classes was the Southeast. Phenolic acids and flavonoids intakes were lower in the North (72.2 and 53.3 mg/d, respectively) and Northeast (82.9 and 56.8 mg/d, respectively).

**Figure 1:**
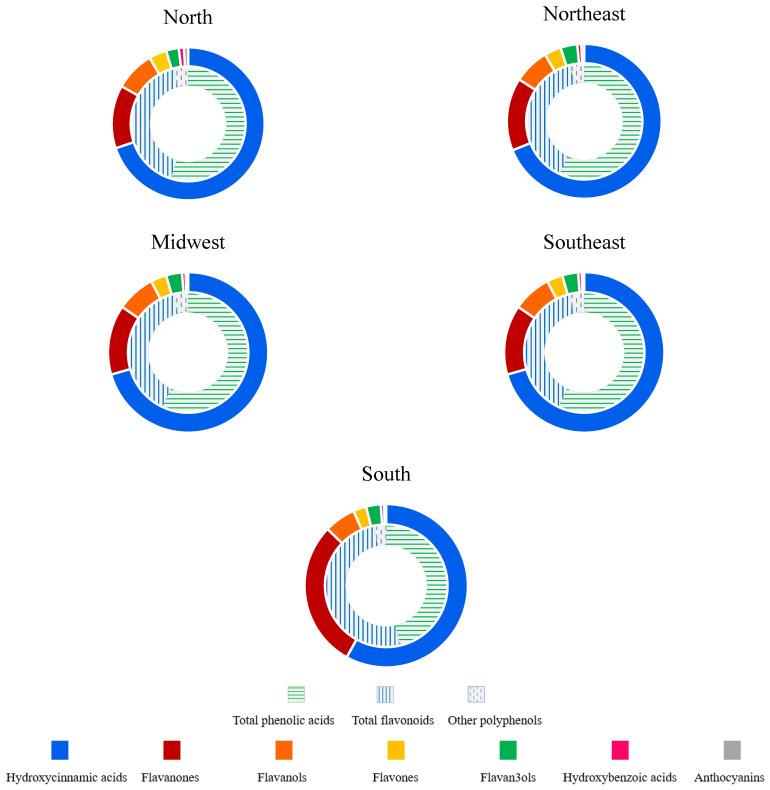
Relative contribution of class and subclass to the total polyphenol intake by Brazilian geographic region. The internal circle represents the percentage of phenolic acids, flavonoids and other polyphenols, while the external circle represents the contribution of each subclass.

Regarding the polyphenol subclasses, hydroxycinnamic acids were the subclass that mostly contributed to total polyphenol intake, with consumption significantly higher (*p* = 0.0001) in the South (108 mg/d) and Southeast (97.1 mg/d) and lower in the North (70.6 mg/d). Flavanones were the second most consumed subclass in all regions, except for the Midwest, for which flavonols were the second main contributor to total polyphenol intake. For all polyphenol classes and subclasses, energy-adjusted intake was higher among women than men, independently of the region (see Supplemental Table S1). Variations in the relative contribution of class and subclass to the total polyphenol were also observed for different Brazilian regions (Figure 1).

**Figure 2:**
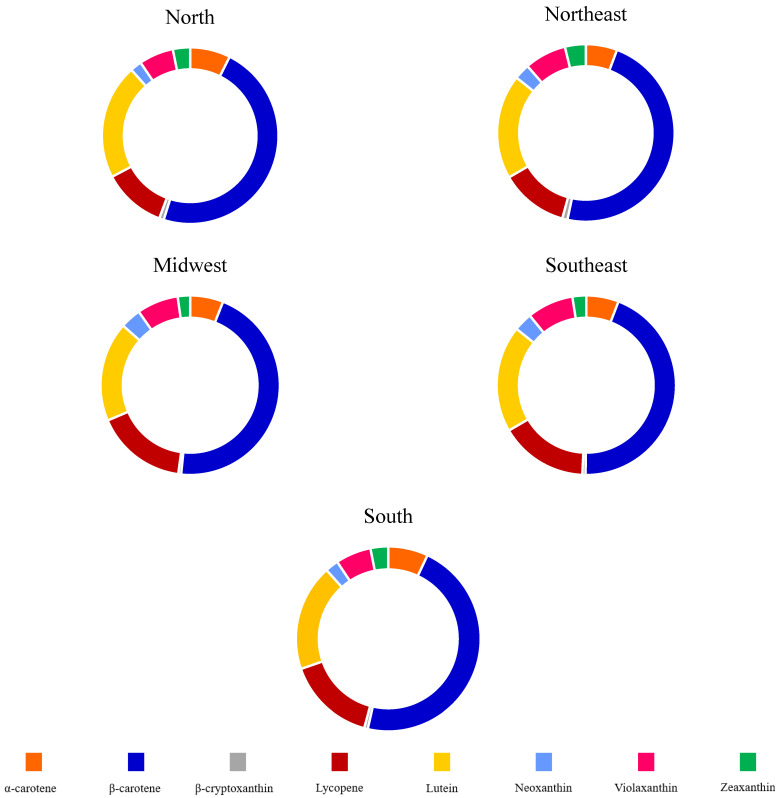
Relative contribution of carotenoids to the total carotenoid intake by Brazilian geographic region.

The total and carotenoid classes’ energy-adjusted intake according to the Brazilian region are presented in Table 3. The consumption of β-carotene, lycopene, neoxanthin, violaxanthin and total carotenoids was significantly higher in Midwest (*p* = 0.0001) compared with other regions (*p* = 0.0001). The region with the lowest energy-adjusted intake of total carotenoid, β-carotene, lycopene, neoxanthin and violaxanthin was North (*p* = 0.001). For all regions, the energy-adjusted total carotenoid and class intake were higher among women than men (see Supplemental Table S2). Despite these differences, β-carotene, lutein and lycopene were the three most consumed carotenoids in all regions (Figure 2).

The main food sources of polyphenols according to the Brazilian region are shown in Table 4. Coffee was the food item that mostly contributed to phenolic acids and hydroxycinnamic acids intake and was the major contributor to total polyphenol consumption in all regions except for the South, in which tea was the food item with a greater contribution to total polyphenol intake. Beans and preparations were the main suppliers of flavonoids and flavonols intake in the Midwest, Southeast, North and Northeast.

The main food contributors to carotenoid intake within each Brazilian region are shown in Table 5. Salads’ contribution to α-carotene, β-carotene, lutein, neoxanthin, violaxanthin and total carotenoids were higher in the Midwest, followed by the Southeast and South, compared to other regions. Açaí, caja juice, mango and mango juice were important suppliers of carotenoid intake in the North and Northeast. Corn and preparations had an important contribution to lutein and zeaxanthin intake, especially in the Northeast.

## 3. Discussion

Brazil is the largest and most populous country in South America. The broad dimension and the influence of Brazilian biomes had a major role in plant species availability and consumption. The influence of the immigrant population in agriculture also reflects in many disparities in dietary patterns in the five Brazilian geographic regions [2,37,38,39,40].

Even though the Brazilian dietary pattern is characterized by rice, beans, flours, oils and caffeinated beverages (called the “traditional pattern”), there are some marked differences in food intake by geographical region [37,41]. Subjects living in the North region presented the lowest adherence to traditional patterns and consumed more regional foods, such as fish, coconut and açaí [37,41,42]. Those living in the Northeast region consumed a high-energy pattern characterized by greater intakes of corn and tuberous roots. The Midwest presented the highest adherence to traditional patterns (greater intake of rice and bean), and the Southeast and South had higher intakes of fruits and vegetables in general [37,42]. As a result of these differences, bioactive compound intake and major food sources presented considerable variations according to region.

The total polyphenol, phenolic acids, flavonoids and other polyphenols energy-adjusted intake was significantly higher in the South compared with other regions, which may be attributable to the greater intakes of polyphenol-dense beverages, mainly tea, coffee, orange juice and wine. Furthermore, the South concentrated the highest average per capita intake for the majority of fruits and vegetables [42]. The Midwest presented higher medians in flavonols intake compared to other regions, which could be due to a greater intake of beans and salads, and the higher intake of anthocyanins by the North may be justified by the higher intake of açaí [42].

The consumption of β-carotene, lycopene, neoxanthin, violaxanthin and total carotenoids was significantly higher in the Midwest, which could be attributable to the greater intake of salads and pumpkin [42]. The South was the second region with the highest consumption of total carotenoids and β-carotene, lycopene and violaxanthin, which is consistent with the highest average per capita intake for many carotenoid sources, such as carrots, tomatoes, oranges, papaya and tangerine [42].

Especially regarding the North and Northeast regions, we highlight some important points. The North region presented the lowest medians in phenolic acids, flavonoids, other polyphenols, β-carotene, lycopene, neoxanthin, violaxanthin and total carotenoid intake compared with other regions. It may be attributable to the reduced average per capita intake of plant-based foods and beverages, such as tea, wine, leafy vegetables (e.g., lettuce, kale and cabbage) and fruits (e.g., banana, orange, apple) [42]. Subjects living in the Northeast also presented lower medians in total polyphenols and class intake compared to other regions, which may be due to lower intake of bioactive-dense food as observed in the North region [42].

In addition to the lower intake of bioactive-dense foods, the North and Northeast concentered the highest prevalence (≥70%) of the inadequacy of calcium, vitamin A, vitamin D and vitamin E intake among males and females of all ages [43]. The North and Northeast had the highest prevalence of households in food insecurity situations, with less than half of the residents with regular access to food [44]. Thus, it is possible to note that the high food biodiversity in the North and Northeast regions does not contribute to better quality diets. The population of these areas consumes insufficient quantities of local plants, probably due to the lack of information on these biodiverse food plants [45,46].

In this sense, we highlight the potential of many native fruits and vegetables, such as açaí, araticum, bacabá, babaçu and cajá-manga, to increase bioactive compound intake in the North and Northeast regions. The systematic investigation of biodiverse food plants, including their availability, consumption and nutritional composition, is a key in the promotion of sustainable diets. In addition, the local and regional utilization of native Brazilian food plants may be an important economic strategy to be explored, considering their potential for sustained explorations under the technological feature. The promotion of the use of native plant species by agricultural producers, as well as the development of new marketable local products, may create a favorable environment to create investment and business opportunities. Besides the economic benefits, it should improve the food insecurity in these regions and contribute to the strengthening of agrifood systems and to the sustainable development of agriculture [4,5,47].

Finally, regarding associations between bioactive compound intake and the risk of chronic diseases, there is no available data concerning this issue across Brazilian geographic regions. A study analyzing National Health Surveys carried out in Brazil in 2008, 2013 and 2019 showed differences in chronic disease prevalence across geographic regions, with cardiovascular diseases and type 2 diabetes being more prevalent in the South, Southeast and Midwest than other regions. Nevertheless, in Brazil, educational level is the main determinant of inequalities in the prevalence of these chronic diseases, and subjects with lower educational levels are less exposed to protective factors, such as healthy eating, physical activity and access to health services than those with higher educational level, independently of the geographic region [48].

In the last years, a growing body of research has been showing the impact of bioactive compound intake on different health outcomes, and systematic reviews and meta-analyses of observational and clinical studies have found protective actions against the risk of chronic diseases [15,49,50,51]. However, it is important to point out that some studies found inverse associations between adverse health outcomes and the intake of specific classes or subclasses, but not total polyphenols or carotenoids [51,52,53]. In this sense, it is fundamental to consider the limitations associated with the estimative of bioactive compound intakes, such as the use of different dietary assessment tools (which differ in their ability to detect the food sources of bioactive compounds) and the lack of analytical data for many foods and compounds [54]. In addition, dietary patterns contributed more than single nutrients or compounds, and this is another limitation that may contribute to these conflicting results [52].

The strengths of our study relate to the use of the most recent data on the Brazilian population’s food intake, in addition to the adoption of a complex sampling design, which allows the extrapolation of results at the population level [42]. Other strengths are the inclusion of the Brazilian food composition data obtained by high-quality analytical methods [55], besides the Phenol-Explorer, providing reliable data. The calculation of polyphenol content in each mixed dish according to the ingredient content, as well as the use of the retention factor, are other strengths. Nevertheless, this study has some limitations. Most food composition data are international, with little information on the polyphenol and carotenoid content in some regional fruits and vegetables consumed in Brazil, especially in the North and Northeast regions.

Considering the importance of this topic, the Brazilian Food Composition Table has been developed with information on food harvested in Brazil; however, it is not comprehensive for all food and compounds [55]. Despite the lack of data representing less than 5% of total plant-based food and beverages intake, the investigation of bioactive compound content in Brazilian food, as well as the comprehensive compilation of this information, should be encouraged to facilitate research on this topic. Another limitation is the lack of information on polyphenol content in the majority of cooked/processed food. Whilst Phenol-Explorer includes retention factor information, it is not comprehensive for all food, compounds and processing methods [56].

In conclusion, our study showed the first detailed analysis of bioactive compound consumption according to Brazilian geographical region. Brazilians living in the South region presented the highest medians of total polyphenols and class intake, with higher intakes of polyphenol-dense foods, such as tea, coffee, orange juice and wine. Regarding carotenoids, the highest medians were found in Midwest and South regions. The highly biodiverse food plants in the North and Northeast regions do not contribute to higher bioactive compound intake, probably due to the lack of information on these plants. Our results point to the need for studies investigating the bioactive composition of native fruits and vegetables by methods of high quality, such as mass spectrometry-based metabolomics, which identifies molecular metabolites and allows investigations on bioactive health effects. In addition, we highlight the potential of many biodiverse food plants for sustained explorations under technological aspects to the development of new marketable products, possibly increasing the consumption of bioactive compounds.

## 4. Methods

### 4.1. Study Population

The data of the present study were retrieved from the NDS 2017–2018. NDS is a cross-sectional survey conducted along with the Household Budget Survey (HBS), a nationwide survey designed to measure Brazilian households’ consumption and expenditures. In summary, HBS adopted a complex sampling plan involving stratified census sectors in two stages. First, the sectors were randomly selected according to their geographical location and socioeconomic classes, and in the second stage, the households were selected by simple random sampling [42]. Of the 57,920 households sampled from the 2017–2018 HBS, 20,112 (34.7%) households were randomly selected for data collection on individual food intake. Thus, the final sample of the 2017–2018 NDS included 46,164 subjects ≥10 years old. The data were collected over a 12-month period to ensure representativeness throughout the year.

### 4.2. Dietary Intake Assessment

Individual food intake data were collected using two non-consecutive 24 h dietary recalls (24 H). Trained researchers conducted personal interviews following sequential stages based on the Multiple-Pass Method [57]. Information regarding foods and beverages consumed during the previous day of the interview, as well as recipes, portion sizes, cooking methods, and time and place of meal were also collected (Supplemental Table S3).

The daily polyphenol and carotenoid intake were estimated based on the two 24 H. To provide more accurate estimates, recipes were converted into ingredients to estimate the amount of all ingredients in each mixed dish cited in the 24 H.

### 4.3. Correspondence between Food Items in Dietary Recalls and in Food Composition Database

The Phenol-Explorer database (www.phenol-explorer.eu/ (accessed on 19 June 2023)) was used to identify the contents of polyphenols in foods. The database encompass data on 501 different polyphenols over 450 foods, which were determined by reverse-phase high-performance liquid chromatography (HPLC), with the exception of proanthocyanins, which were determined by normal-phase HPLC. In the case of foods containing polyphenols linked to the food matrix that cannot be released under normal extraction conditions, data were obtained by HPLC after basic or acid hydrolysis [58].

For some regional foods and beverages (e.g., açai and yerba mate tea) and other items highly prevalent in the Brazilian diet (e.g., rice, beans and orange), we used data from food collected and analyzed in Brazil by HPLC. We also used data from the Brazilian Food Composition Database (TBCA, available at www.tbca.net.br/ (accessed on 19 June 2023)), an online database with data on flavonoid content (expressed as aglycone) identified and analyzed using HPLC [58]. In the case of overlapping data between Brazilian data and the Phenol-Explorer database, Brazilian data had priority.

Polyphenol intake was expressed as aglycone equivalents, and the glycosides and esters polyphenols were converted by removing the contribution to the molecular weight of the non-phenolic part of the molecule. In order to supply reliable data on dietary polyphenol intake, the effects of processing methods on polyphenol content were considered using retention factors (RF) from the Phenol-Explorer database [56].

The carotenoid concentration (α-carotene, β-carotene, β-cryptoxanthin, lycopene, lutein, neoxanthin, violaxanthin and zeaxanthin) of food consumed was obtained from the literature. The priority was to use data from food harvested and analyzed in Brazil by high-performance liquid chromatography (HPLC). We also used data from TBCA, which contains data on the carotenoid content of Brazilian food analyzed by HPLC [40]. In the case in which the carotenoid composition was not available for food collected in Brazil, the data were obtained from HPLC analysis from Spain (avocado, asparagus, beetroot, onion and apricot), Argentina (chard), China (persimmon), Indonesia (cashew nut), Iran (saffron), Italy (grape), Mexico (egg) and Portugal (cherry). Information collected on carotenoid concentrations was those correspond to raw or cooked foods, depending on how they were consumed.

The main polyphenols and carotenoids of food consumed in NDS survey with content described are reported in Supplemental Table S4.

### 4.4. Estimation of Intake and Dietary Contributors

Since food composition data are reported in mg/100 g, the individual polyphenol and carotenoid intake from each food was calculated by multiplying the content of each compound by the daily amount of each food consumed and dividing by 100. Total classes and subclasses of polyphenol intake, as well as total and carotenoid classes, were calculated by summing up intakes from all food sources consumed. The main food contributors to total and classes of polyphenols and carotenoid intake were determined by the percentage of contribution to the median daily intake.

### 4.5. Demographic Information

Demographic variables were collected by trained researchers in personal interviews using a structured questionnaire and included data regarding sex, age, race, Brazilian region and household area (urban or rural). The Brazilian regions consisted of five categories: North (states of Acre, Amapá, Amazonas, Pará, Rondônia, Roraima and Tocantins), Northeast (states of Alagoas, Bahia, Ceará, Maranhão, Paraíba, Piauí, Pernambuco, Rio Grande do Norte and Sergipe), Middle-West (states of Goiás, Mato Grosso and Mato Grosso do Sul), Southeast (states of Espírito Santo, Minas Gerais, Rio de Janeiro and São Paulo) and South (states of Paraná, Rio Grande do Sul and Santa Catarina).

### 4.6. Statistical Analysis

Data were presented in medians for continuous variables and frequencies or percentages for categorical variables. Polyphenol and carotenoid intake data from the two 24 H were statistically adjusted for the usual intake distribution and removal of intrapersonal variation using the statistical technique Multiple Source Method. The polyphenol and carotenoid intake distribution was analyzed using the Kolmogorov–Smirnov test, and it did not follow a normal distribution. Thus, the intakes were presented as medians and 25th and 75th percentiles, and the Kruskal–Wallis test was used to test differences according to Brazilian regions (*p* values < 0.05 were considered significant). Since bioactive compound intake increases according to higher food consumption, the polyphenol and carotenoid intake was also calculated in energy-adjusted terms (mg per 1000 kcal per day (4184 kJ/d) of total energy consumed). Analyses were performed using sample weights to allow population representativeness using Stata software version 17.

## Figures and Tables

**Table 1:** Socio-economic and demographic characteristics of the studied population.

Characteristics	North		Northeast		Midwest		Southeast		South	
	n	%	n	%	n	%	n	%	n	%
Total	6836	14.8	16,097	34.9	5740	12.4	11,471	24.9	6020	13.0
Sex										
Men	3244	47.5	7285	45.3	2783	48.5	5327	46.4	2821	46.9
Women	3592	52.5	8812	54.7	2957	51.5	6144	53.6	3199	53.1
Age group (years)										
10–13	653	9.6	1178	7.3	425	7.4	692	6.0	323	5.4
14–18	800	11.7	1633	10.1	560	9.8	943	8.2	471	7.8
19–59	4534	66.3	10,447	64.9	3803	66.3	7449	64.9	3917	65.1
≥60	849	12.4	2839	17.6	952	16.6	2387	20.8	1309	21.7
Race/ethnicity										
White	1216	17.8	4185	26.0	1987	34.6	5327	46.4	4489	74.6
Others	5620	82.2	11,912	74.0	3753	65.4	6144	53.6	1531	25.4
Household situation										
Urban	4764	69.7	12,932	80.3	3828	66.7	9418	82.1	4449	73.9
Rural	2072	30.3	3165	19.7	1912	33.3	2053	17.9	1571	26.1
Per capita income (PPP) *	$497.7 ± 14.8		$552.0 ± 7.5		$1156.5 ± 22.7		$1017.7 ± 12.7		$1046.8 ± 16.1	

* Mean ± standard error of the mean.

**Table 2:** Energy-adjusted total and polyphenol class and subclass intake (mg/1000 kcal/d) according to Brazilian geographic region.

Polyphenols	North		Northeast		Midwest		Southeast		South		*p* *
	Median	25–75th Percentiles	Median	25–75th Percentiles	Median	25–75th Percentiles	Median	25–75th Percentiles	Median	25–75th Percentiles	
Phenolic acids	72.2	46.1–119.6	82.9	49.1–134.7	85.4	46.9–149.5	99.3	54.6–159.2	110.3	60.4–207.0	0.0001
Hydroxyenzoic acids	1.1	0.6–2.1	1.0	0.6–2.4	1.0	0.6–2.2	1.0	0.6–2.2	1.2	0.6–2.8	0.0001
Hydroxycinnamic acids	70.6	43.9–117.2	80.6	46.7–132.1	83.5	44.5–146.7	97.1	52.7–156.1	108.0	58.0–203.3	0.0001
Flavonoids	53.5	26.2–283.9	56.8	25.4–252.9	57.9	26.4–223.8	67.6	29.6–352.6	119.6	43.1–529.7	0.0001
Flavan3ols	2.7	1.0–9.7	4.1	1.2–13.1	3.7	0.9–14.2	4.4	1.2–16.4	5.4	1.4–18.9	0.0001
Flavones	3.8	2.3–5.4	4.1	2.5–5.9	3.7	2.3–5.3	4.4	3.0–6.0	4.8	3.3–6.6	0.0001
Flavonols	8.5	5.5–12.4	8.7	5.9–12.6	11.9	8.2–17.0	10.7	7.5–15.5	11.6	7.2–21.1	0.0001
Flavanones	13.5	2.2–236.5	17.8	3.0–201.9	9.8	3.4–116.9	19.3	3.7–291.0	54.3	5.7–472.4	0.0001
Anthocyanins	0.9	0.4–5.1	0.7	0.3–1.9	0.7	0.3–1.9	0.7	0.3–1.7	0.8	0.3–2.3	0.0001
Other polyphenols ^†^	5.8	4.2–8.1	5.8	4.2–8.0	6.2	4.4–8.9	7.2	5.2–10.2	7.8	5.3–11.5	0.0001
Total polyphenols	214.6	123.5–446.2	195.2	120.3–416.5	207.2	122.5–432.7	236.4	145.0–521.8	353.3	173.9–734.1	0.0001

Estimates were performed using sample weights to allow population representativeness. * Comparisons across categories were performed using the Kruskal–Wallis test. ^†^ Other polyphenols as the sum of lignans, stilbenes, alkylphenols, alkylmethoxyphenols, methoxyphenols, furanocoumarins, hydroxybenzaldehydes, hydroxycoumarins, tyrosols, catechol, phenol, pyrogallol and arbutin.

**Table 3:** Energy-adjusted total and carotenoid class (mg/1000 kcal/d) according to Brazilian geographic region.

Polyphenols	North		Northeast		Midwest		Southeast		South		*p* *
	Median	25–75th Percentiles	Median	25–75th Percentiles	Median	25–75th Percentiles	Median	25–75th Percentiles	Median	25–75th Percentiles	
α-carotene	0.7	0.3–2.6	0.6	0.2–2.0	0.8	0.3–1.9	0.7	0.3–1.9	0.9	0.3–2.3	0.0001
β-carotene	4.5	1.6–12.1	5.0	1.9–11.2	6.1	2.5–10.9	5.3	2.3–10.5	6.0	2.5–12.3	0.0001
β-cryptoxanthin	0.08	0.0–0.2	0.1	0.0–0.2	0.07	0.0–0.2	0.07	0.0–0.2	0.08	0.0–0.2	0.0001
Lycopene	1.1	0.5–2.5	1.3	0.6–2.8	2.2	1.1–3.6	1.9	0.9–3.2	2.0	0.8–3.5	0.0001
Lutein	2.0	0.9–3.5	2.0	1.1–3.7	2.4	1.2–4.0	2.3	1.3–4.1	2.4	1.4–4.2	0.0001
Neoxanthin	0.2	0.0–0.6	0.3	0.1–0.6	0.5	0.2–0.9	0.4	0.2–0.8	0.3	0.1–0.8	0.0001
Violaxanthin	0.6	0.3–1.5	0.8	0.3–1.9	1.0	0.5–2.0	1.0	0.5–1.9	0.8	0.4–1.8	0.0001
Zeaxanthin	0.3	0.2–0.5	0.4	0.2–0.6	0.3	0.2–0.4	0.3	0.2–0.5	0.4	0.2–0.5	0.0001
Total carotenoids	12.3	6.2–25.2	13.3	7.2–24.0	15.3	8.3–25.0	13.7	7.8–23.1	15.1	8.3–26.5	0.0001

Estimates were performed using sample weights to allow population representativeness. * Comparisons across categories were performed using the Kruskal–Wallis test.

**Table 4:** The main food sources of polyphenols according to Brazilian geographic region.

Polyphenol Classes and Subclasses	Rank	North		Northeast		Midwest		Southeast		South	
		Food Item	%	Food Item	%	Food Item	%	Food Item	%	Food Item	%
Phenolic acids	1	Coffee	84.8	Coffee	85.2	Coffee	84.3	Coffee	86.1	Coffee	71.3
	2	Rice and preparations	4.0	Rice and preparations	4.4	Rice and preparations	6.0	Rice and preparations	3.2	Tea	12.1
	3	Açaí	1.6	Bread	0.7	Potato	0.6	Bread	0.9	Rice and preparations	2.1
Hydroxybenzoic acids	1	Beer	9.8	Beer	9.9	Beer	13.4	Tea	25.2	Tea	84.4
	2	Rice and preparations	6.8	Rice and preparations	7.2	Tea	11.7	Beer	11.7	Wine	2.5
	3	Açaí	7.8	Banana	1.9	Rice and preparations	9.9	Rice and preparations	3.2	Beer	1.5
Hydroxycinnamic acids	1	Coffee	86.3	Coffee	86.4	Coffee	86.2	Coffee	87.5	Coffee	82.0
	2	Rice and preparations	4.0	Rice and preparations	4.3	Rice and preparations	4.9	Rice and preparations	3.1	Rice and preparations	2.3
	3	Açaí	1.3	Bread	0.7	Potato	0.6	Bread	0.9	Tea	1.9
Flavonoids	1	Bean and preparations	40.7	Bean and preparations	46.9	Bean and preparations	47.6	Bean and preparations	45.0	Tea	52.2
	2	Orange juice	15.1	Orange juice	12.8	Orange juice	16.5	Orange juice	11.2	Bean and preparations	15.6
	3	Bread and sandwiches	2.9	Bread and sandwiches	3.8	Tea	5.2	Tea	3.2	Orange juice	6.5
Flavan3ols	1	Chocolate	7.0	Chocolate	9.8	Tea	16.6	Tea	24.6	Tea	84.5
	2	Chocolate powder	2.8	Chocolate powder	2.3	Chocolate	10.4	Chocolate	12.6	Chocolate	14.2
	3	Tucumã’s bread	1.4	Grape	1.1	Chocolate powder	5.5	Chocolate powder	5.1	Wine	5.4
Flavones	1	Bread and sandwiches	47.8	Bread and sandwiches	36.8	Pasta and preparations	31.5	Bread and sandwiches	44.4	Pasta and preparations	40.9
	2	Pasta and preparations	23.5	Pasta and preparations	28.1	Bread and sandwiches	24.3	Pasta and preparations	34.4	Bread and sandwiches	26.1
	3	Cracker	4.3	Cracker	7.4	Cracker	4.2	Cracker	3.7	Cracker	2.6
Flavonols	1	Bean and preparations	52.9	Bean and preparations	58.1	Bean and preparations	60.6	Bean and preparations	55.9	Tea	44.4
	2	Salads	6.0	Salads	6.8	Salads	11.3	Salads	9.3	Bean and preparations	27.0
	3	Soups and broths	5.0	Apple	4.8	Tea	7.8	Apple	7.5	Salads	3.5
Flavanones	1	Orange juice	81.2	Orange juice	83.8	Orange juice	76.2	Orange juice	82.9	Orange juice	80.7
	2	Orange	11.9	Orange	15.6	Orange	13.4	Orange	13.7	Orange	17.8
	3	Lemon	0.2	Lemon	0.4	Lemon	0.4	Fruit salad	0.9	Fruit salad	1.1
Anthocyanins	1	Açaí	51.2	Açaí	31.9	Bean and preparations	17.2	Bean and preparations	17.0	Grape juice	21.3
	2	Bean and preparations	13.1	Bean and preparations	25.1	Açaí	6.1	Grape juice	12.3	Bean and preparations	15.3
	3	Bean’s soup	2.0	Grape	1.9	Grape juice	5.5	Açaí	4.2	Wine	3.2
Other	1	Coffee	25.3	Coffee	28.9	Coffee	20.6	Coffee	17.8	Coffee	17.4
	2	Wheat flour products	8.4	Orange juice	17.7	Wheat flour products	6.9	Wheat flour products	6.5	Orange juice	15.4
	3	Açaí	4.7	Wheat flour products	14.3	Orange juice	5.3	Orange juice	6.2	Wheat flour products	13.7
Total polyphenols	1	Coffee	57.3	Coffee	57.0	Coffee	54.3	Coffee	56.0	Tea	35.0
	2	Bean and preparations	8.3	Bean and preparations	13.6	Bean and preparations	17.8	Bean and preparations	15.1	Coffee	33.7
	3	Orange juice	6.2	Orange juice	5.4	Orange juice	5.0	Orange juice	5.1	Bean and preparations	7.4

**Table 5:** The main food sources of carotenoids according to Brazilian geographic region.

Carotenoid	Rank	North		Northeast		Midwest		Southeast		South	
		Food Item	%	Food Item	%	Food Item	%	Food Item	%	Food Item	%
α-carotene	1	Soups and broths	14.1	Salads	10.8	Salad	23.8	Salads	13.4	Salads	11.2
	2	Salads	7.9	Pumpkin	6.9	Pumpkin	19.6	Pumpkin	12.4	Carrot	8.1
	3	Banana	2.1	Carrot	2.0	Carrot	6.3	Carrot	8.2	Banana	5.6
β-carotene	1	Açaí	6.8	Pumpkin	7.8	Salads	26.2	Salads	18.4	Salads	15.7
	2	Salads	5.8	Salads	6.7	Pumpkin	9.8	Pumpkin	5.7	Carrot	5.5
	3	Pumpkin	1.9	Maracuja juice	3.2	Carrot	4.5	Carrot	5.6	Pumpkin	1.9
β-cryptoxanthin	1	Caja juice	12.6	Papaya	11.5	Tangerine	14.4	Papaya	10.1	Tangerine	33.4
	2	Orange juice	11.9	Maracuja juice	10.0	Orange juice	10.5	Orange juice	9.3	Orange juice	17.4
	3	Papaya	5.0	Caja juice	5.0	Papaya	7.1	Fruit salad	5.8	Papaya	15.7
Lycopene	1	Tomato	8.5	Tomato sauce	19.9	Tomato	23.4	Tomato	20.4	Tomato	25.2
	2	Watermelon	4.1	Tomato	9.2	Tomato sauce	9.9	Tomato sauce	16.9	Tomato sauce	22.6
	3	Papaya	2.7	Papaya	5.2	Papaya	3.9	Papaya	4.2	Papaya	5.4
Lutein	1	Açaí	6.7	Corn and preparations	22.1	Salads	23.6	Salads	18.2	Salad	17.7
	2	Salad	5.9	Salads	6.1	Corn and preparations	6.1	Corn and preparations	5.9	Corn and preparations	10.8
	3	Egg	5.2	Eggs	6.0	Eggs	3.0	Eggs	5.1	Eggs	3.8
Neoxanthin	1	Salads	15.3	Salads	19.1	Salads	38.0	Salads	32.8	Salads	28.2
	2	Mango	5.2	Corn and preparations	10.3	Mango	4.1	Kale	5.4	Kale	4.1
	3	Mango juice	3.9	Mango	6.2	Lettuce	3.5	Mango	2.8	Lettuce	3.9
Violaxanthin	1	Mango juice	14.4	Salads	13.6	Salads	33.1	Salads	32.1	Salads	28.0
	2	Salads	10.6	Mango juice	12.6	Mango	6.2	Mango	4.2	Mango	4.0
	3	Mango	9.7	Mango	10.1	Mango juice	4.1	Mango juice	3.2	Mango juice	3.2
Zeaxanthin	1	Corn and preparations	12.1	Corn and preparations	54.1	Corn and preparations	27.4	Corn and preparations	23.1	Corn and preparations	31.1
	2	Orange juice	10.5	Orange juice	12.5	Orange juice	8.2	Orange juice	5.7	Orange juice	13.9
	3	Mango	7.7	Mango	6.9	Orange	2.3	Orange	2.3	Orange	3.1
Total carotenoids	1	Salads	8.2	Salads	9.1	Salads	23.2	Salads	19.1	Salads	16.8
	2	Açaí	5.8	Corn and preparations	8.3	Pumpkin	7.1	Pumpkin	5.4	Tomato	4.5
	3	Pumpkin	1.3	Pumpkin	5.2	Tomato	5.0	Tomato	4.9	Pumpkin	3.1

## Data Availability

The HBS survey is carried out by the Brazilian Institute of Geography and Statistics (IBGE) and the Brazilian Ministry of Health. The research complies with the Brazilian Federal Law (number 5534 of 14 November 1968), which presents the confidential nature of research conducted by the IBGE. Thus, the ethical review and approval of this study were waived since the data are secondary and public, without identification of the subject, address or telephone number.

## Supplementary Materials

The following are available online at https://www.mdpi.com/article/10.3390/plants12132414/s1, Supplemental Table S1. Energy-adjusted total and polyphenols class and subclass intake (mg/1000 kcal/d) according to Brazilian geographic region and sex. Supplemental Table S2. Energy-adjusted total and carotenoid class (mg/1000 kcal/d) according to Brazilian geographic region and sex. Supplemental Table S3. The 24-h dietary recall used in the NDS survey. Supplemental Table S4. Polyphenols and carotenoids profile of foods consumed in NDS survey.
