# Supplementary material for: Bioactive Compounds Intake of the Brazilian Population According to Geographic Region
Source: Plants (Basel). 2023 Jun 22;12(13):2414. doi: 10.3390/plants12132414 (PMC10346977; doi:10.3390/plants12132414)
Supplement: Supplementary file 1 [file plants-12-02414-s001.zip › plants-2418728-supplementary.pdf]

Supplemental Table S1. Energy-adjusted total and polyphenols class and subclass intake (mg/ 1000kcal/ d) according to Brazilian geographic region and sex.

Polyphenols	North		Northeast		Midwest		Southeast		South	
	Median	25-75th percentiles	Median	25-75th percentiles	Median	25-75th percentiles	Median	25-75th percentiles	Median	25-75th percentiles
Phenolic acids										
Men	70.4	44.4-114.3	77.6	46.7-126.9	77.9	42.8-140.0	94.1	52.4-151.0	98.6	53.9-184.9
Women	74.5	47.4-127.5	87.5	51.7-140.2	91.4	51.2-155.9	103.4	56.8-164.7	123.5	65.7-226.7
P	0.0004		0.0001		0.0001		0.0001		0.0001	
Hydroxyenzoic acids										
Men	1.0	0.6-1.9	0.9	0.6-2.0	1.0	0.6-1.8	0.9	0.5-1.9	1.0	0.6-2.2
Women	1.2	0.6-2.3	1.2	0.6-2.3	1.1	0.7-2.5	1.1	0.6-2.5	1.4	0.7-3.3
P	0.0001		0.0001		0.0001		0.0001		0.0001	
Hydroxycinnamic acids										
Men	68.7	42.6-111.8	75.6	44.5-125.2	75.9	41.4-138.3	92.6	50.9-148.8	97.0	52.1-182.8
Women	72.1	45.2-125.8	84.2	48.9-137.3	89.5	48.8-152.4	100.8	54.4-160.9	120.3	63.4-223.2
P	0.0011		0.0001		0.0001		0.0001		0.0001	
Flavonoids										
Men	45.2	23.3-236.3	47.9	22.8-209.8	48.9	24.0-207.1	56.5	27.2-279.5	97.1	36.4-485.4
Women	60.2	28.9-340.7	63.9	28.5-291.1	67.9	28.3-237.9	77.6	32.2-412.5	144.0	51.3-564.5
P	0.0001		0.0001		0.0001		0.0001		0.0001	
Flavan3ols										

Men	2.2	0.8-7.6	3.3	0.9-10.7	2.9	0.7-11.9	3.5	1.0-13.1	4.1	1.0-14.2
Women	3.3	1.2-12.0	4.9	1.4-15.2	4.4	1.0-16.5	5.2	1.5-19.7	7.3	1.9-23.2
P	0.0001		0.0001		0.0001		0.0001		0.0001	
Flavones										
Men	3.5	2.1-5.2	4.0	2.3-5.7	3.5	2.0-4.6	4.3	2.8-5.8	4.6	3.1-6.4
Women	3.9	2.5-5.5	4.3	2.7-6.1	4.0	2.5-5.8	4.6	3.2-6.2	5.0	3.5-6.7
P	0.0001		0.0001		0.0001		0.0001		0.0001	
Flavonols										
Men	8.3	5.4-11.8	8.3	5.8-12.0	11.5	8.0-15.5	10.1	7.3-14.4	10.4	6.7-17.9
Women	8.8	5.7-13.1	9.0	6.1-13.2	12.3	8.3-18.4	11.1	7.7-16.4	13.4	7.8-23.7
P	0.0001		0.0001		0.0001		0.0001		0.0001	
Flavanones										
Men	11.2	2.0-191.1	14.1	2.6-159.9	9.2	2.9-118.4	15.5	3.4-221.1	45.9	5.0-445.3
Women	15.2	2.4-276.9	22.2	3.4-241.9	10.9	3.8-114.0	26.2	4.0-347.4	63.5	6.3-501.8
P	0.0001		0.0001		0.0001		0.0001		0.0001	
Anthocyanins										
Men	0.7	0.3-3.5	0.6	0.3-1.7	0.6	0.3-1.5	0.6	0.3-1.5	0.6	0.3-1.7
Women	1.0	0.4-7.1	0.8	0.3-2.1	0.8	0.3-2.4	0.8	0.4-2.0	0.9	0.4-3.0
P	0.0001		0.0001		0.0001		0.0001		0.0001	
Other polyphenols[†]										
Men	5.6	4.1-7.7	5.5	4.1-7.6	5.8	4.2-8.4	6.9	5.0-9.5	7.4	5.0-10.6
Women	6.0	4.4-8.5	6.0	4.4-8.4	6.5	4.6-9.4	7.6	5.4-10.8	8.3	5.6-12.4

P		0.0001		0.0001		0.0001		0.0001		0.0001
Total polyphenols										
Men	195.5	113.1-406.4	177.7	111.6-368.7	190.8	113.0-405.7	212.9	135.4-451.0	309.3	150.4-662.2
Women	233.6	136.3-492.1	210.7	129.1-457.4	217.7	132.3-462.6	257.7	156.2-581.2	394.1	200.8-797.7
P		0.0001		0.0001		0.0001		0.0001		0.0001

Estimates were performed using sample weights to allow population representativeness.

*Comparisons across categories were performed by using the Kruskal-Wallis test.

†Other polyphenols as the sum of lignans, stilbenes, alkylphenols, alkylmethoxyphenols, methoxyphenols, furanocoumarins, hydroxybenzaldehydes, hydroxycoumarins, tyrosols, catechol, phenol, pyrogallol and arbutin.

Supplemental Table S2. Energy-adjusted total and carotenoid class (mg/ 1000kcal/ d) according to Brazilian geographic region and sex.

Polyphenols	North		Northeast		Midwest		Southeast		South	
	Median	25-75th percentiles	Median	25-75th percentiles	Median	25-75th percentiles	Median	25-75th percentiles	Median	25-75th percentiles
α-carotene										
Men	0.7	0.2-2.4	0.5	0.2-1.6	0.7	0.2-1.9	0.6	0.2-1.7	0.8	0.3-2.0
Women	0.8	0.3-2.8	0.8	0.3-2.3	1.0	0.3-2.1	0.8	0.3-2.1	1.0	0.4-2.6
P	0.0001		0.0001		0.0001		0.0001		0.0001	
β-carotene										
Men	4.0	1.3-11.0	4.2	1.6-9.5	5.0	1.9-8.9	4.4	1.8-8.8	5.0	2.1-10.5
Women	5.0	1.8-13.2	5.9	2.3-12.4	7.2	3.4-12.9	6.2	2.7-11.9	7.0	3.0-12.9
P	0.0001		0.0001		0.0001		0.0001		0.0001	
β-cryptoxanthin										
Men	0.07	0.03-0.2	0.1	0.04-0.2	0.05	0.03-0.1	0.06	0.03-0.1	0.07	0.03-0.2
Women	1.0	0.05-0.2	0.1	0.05-0.3	0.08	0.04-0.2	0.08	0.04-0.2	1.0	0.05-0.2
P	0.0001		0.0001		0.0001		0.0001		0.0001	
Lycopene										
Men	1.0	0.5-2.2	1.1	0.5-2.3	1.8	0.9-3.0	1.7	0.7-2.9	1.8	0.7-3.2
Women	1.3	0.6-2.7	1.5	0.7-3.1	2.5	1.3-3.9	2.1	1.0-3.4	2.2	1.0-3.7
P	0.0001		0.0001		0.0001		0.0001		0.0001	
Lutein										
Men	1.7	0.8-3.3	1.8	1.0-3.3	2.0	1.0-3.3	2.0	1.1-3.6	2.1	1.2-3.7

Women	2.1	1.0-3.7	2.2	1.2-3.9	2.8	1.5-4.6	2.6	1.4-4.5	2.8	1.6-4.6
P		0.0001		0.0001		0.0001		0.0001		0.0001
Neoxanthin										
Men	0.2	0.1-0.5	0.2	0.1-0.5	0.4	0.1-0.7	0.3	0.1-0.7	0.3	0.1-0.7
Women	0.3	0.1-0.7	0.3	0.1-0.7	0.5	0.2-1.1	0.5	0.2-0.9	0.4	0.2-0.9
P		0.0001		0.0001		0.0001		0.0001		0.0001
Violaxanthin										
Men	0.6	0.2-1.4	0.7	0.3-1.7	0.8	0.4-1.6	0.9	0.4-1.7	0.7	0.3-1.6
Women	0.7	0.3-1.7	0.9	0.4-2.1	1.2	0.5-2.5	1.1	0.5-2.1	1.0	0.5-2.0
P		0.0001		0.0001		0.0001		0.0001		0.0001
Zeaxanthin										
Men	0.2	0.1-0.5	0.3	0.2-0.6	0.2	0.1-0.4	0.2	0.1-0.4	0.3	0.2-0.5
Women	0.3	0.2-0.5	0.4	0.2-0.6	0.3	0.2-0.5	0.3	0.2-0.5	0.4	0.2-0.5
P		0.0001		0.0001		0.0001		0.0001		0.0001
Total carotenoids										
Men	11.2	5.3-22.4	11.4	6.2-21.1	12.6	6.7-20.0	12.0	6.8-20.0	13.2	7.2-22.6
Women	13.5	7.2-27.4	15.0	8.4-26.5	17.4	10.1-28.0	15.6	8.9-25.9	17.1	9.6-30.0
P		0.0001		0.0001		0.0001		0.0001		0.0001

Estimates were performed using sample weights to allow population representativeness.

*Comparisons across categories were performed by using the Kruskal-Wallis test.

Supplemental Table S3. The 24-hour dietary recall used in the NDS survey.

DAY OF WEEK:					
Quick list	Time	Description of food and drink (including amount and cooking method)	Occasion of consumption code	Place of consumption code	Adition

Supplemental Table S4. Polyphenols and carotenoids profile of foods consumed in NDS survey.

Food	Scientific name	Polyphenols	Carotenoids
**Fruits**			
Abiu	*Pouteria caimito*	Caffeic acid, 5-caffeoylquinic acid, ferulic acid, p-coumaric acid, quercetin, apigenin	β-carotene
Açai	*Euterpe oleraceae Mart.*	Cyanidin 3-glucoside, cyanidin 3-sambubioside, cyanidin 3-rutinoside, peonidin 3-glucoside, peonidin 3-rutinoside, procyanidin B1	α-carotene, β-carotene, lutein
Acerola	*Malpighia emarginata*	Protocatechuic acid, vanillic acid, -caffeoylquinic acid, p-coumaric acid, kaempferol, catechin, epicatechin, cyanidin, pelargonidin	α-carotene, β-carotene, β-cryptoxanthin, lutein, neoxanthin, violaxanthin, zeaxanthin
Apple	*Malus domestica*	Quercetin, catechin, epicatechin	β-carotene, lutein
Apricot	*Prunus armeniaca L.*	3-Caffeoylquinic acid, 5-caffeoylquinic acid, 3-feruloylquinic acid, 5-feruloylquinic acid, 3-p-coumaroylquinic acid, 5-p-coumaroylquinic acid, quercetin, quercetin 3-O-rutinoside, kaempferol 3-O-rutinoside, catechin, epicatechin, procyanidin dimers (B1, B3, B7) procyanidin trimer EEC, lariciresinol, matairesinol, pinoresinol, secoisolariciresinol	β-carotene, β-cryptoxanthin
Araçá	*Psidium cattleyanum*	Ferulic acid, p-coumaric acid, quercetin, kaempferol	α-carotene, β-carotene, β-cryptoxanthin, lutein, zeaxanthin
Avocado	*Persea americana Mill.*	Epicatechin, epicatechin 3-O-gallate, lariciresinol, matairesinol, medioresinol, pinoresinol, secoisolariciresinol, syringaresinol, procyanidin dimer B2	α-carotene, β-carotene, lutein
Bacabá	*Oenocarpus bacaba Mart.*	Protocatechuic acid, vanillic acid, caffeic acid, 5-caffeoylquinic acid, cinnamic acid, ferulic acid, p-coumaric acid, quercetin 3-O-glucoside, naringenin, eriodictyol, vanillin	-
Banana	*Musa acuminata (prata)*	Gallic acid, catechin, epicatechin, epigallocatechin, prodelphinidin dimer B3, lariciresinol, matairesinol, pinoresinol, secoisolariciresinol, syringaresinol	α-carotene, β-carotene, lutein
Biribá	*Rollinia mucosa (Jacq.) Baill*	Ferulic acid, apigenin	-
Blackberry	*Rubus ulmifolius*	4-Hydroxybenzoic acid 4-O-glucoside, ellagic acid, gallic acid, galloyl glucose, protocatechuic acid 4-O-glucoside, caffeic acid, 3-caffeoylquinic acid, 4-caffeoylquinic acid, 5-caffeoylquinic acid, caffeoyl glucose, ferulic acid, 3-feruloylquinic acid, feruloyl glucose, 3-p-coumaroylquinic acid, p-coumaroyl glucose, p-coumaric acid, p-coumaric acid 4-O-glucoside, quercetin, quercetin 3-O-glucoside, quercetin 3-O-rutinoside, quercetin 3-O-galactoside, quercetin 3-O-glucosyl-xyloside, quercetin 3-O-xyloside, quercetin 3-O-xylosyl-glucuronide, myricetin, kaempferol, catechin, epicatechin, epigallocatechin, cyanidin 3-O-glucoside, cyanidin 3-O-rutinoside, cyanidin 3-O-xyloside, cyanidin 3-O-(6-malonyl-glucoside), cyanidin 3-O-(6-dioxalyl-glucoside), procyanidin dimers (B1, B2, B3), procyanidin trimer EEC, prodelphinidin	α-carotene, β-carotene

		dimer B3, lariciresinol, matairesinol, pinoresinol, secoisolariciresinol, syringaresinol, cyclolariciresinol	
Buriti	*Mauritia flexuosa*	Quercetin	α-carotene, β-carotene
Butiá	*Butia capitata*	Catechin	α-carotene, β-carotene
Cajá	*Spondias mombin*	-	α-carotene, β-carotene, β-cryptoxanthin, lutein
Cashew	*Anacardium occidentale*	Quercetin 3-O-glucoside, quercetin 3-O-galactoside, quercetin 3-O-rhamnoside, quercetin 3-O-xylopyranoside, quercetin 3-O-arabinopyrannoside, quercetin 3-O-arabinofuranoside, myricetin 3-O-glucoside, myricetin 3-O-galactoside, myricetin 3-O-xylopyranoside, myricetin 3-O-arabinopyrannoside, myricetin 3-O-arabinofuranoside, myricetin 3-O-rhamnoside	α-carotene, β-carotene, β-cryptoxanthin, lutein, violaxanthin, zeaxanthin
Cherry	*Prunus avium L.*	3-Caffeoylquinic acid, 4-caffeoylquinic acid, 5-caffeoylquinic acid, 3-feruloylquinic acid, 3-p-coumaroylquinic acid, 4-p-coumaroylquinic acid, quercetin, catechin, epicatechin, epicatechin 3-O-gallate, epigallocatechin, cyanidin 3-O-glucoside, cyanidin 3-O-rutinoside, peonidin 3-O-rutinoside, peonidin 3-O-glucoside, pelargonidin 3-O-rutinoside, procyanidin dimers (B1, B2, B3, B4, B5, B7), procyanidin trimer C1	α-carotene, β-carotene, β-cryptoxanthin, lutein, violaxanthin, zeaxanthin
Coco ouricuri	*Syagrus coronata (Martius) Beccari*	-	β-carotene, β-cryptoxanthin, lycopene
Coco pupunha	*Bactris gasipaes*	5-Caffeoylquinic acid, protocatechuic acid, ferulic acid, p-coumaric acid, myricetin, apigenin	α-carotene, β-carotene, β-cryptoxanthin, lycopene
Cranberry	*Vaccinium macrocarpon Aiton*	2-Hydroxybenzoic acid, 3-hydroxybenzoic acid, 4-hydroxybenzoic acid, 2,3-dihydroxybenzoic acid, 2,4-dihydroxybenzoic acid, benzoic acid, vanillic acid, caffeic acid, cinnamic acid, ferulic acid, o-coumaric acid, p-coumaric acid, sinapic acid, 4-hydroxyphenylacetic acid, quercetin, quercetin 3-O-galactoside, quercetin 3-O-arabinoside, quercetin 3-O-rhamnoside, myricetin, myricetin 3-O-arabinoside, kaempferol, kaempferol 3-O-glucoside, cyanidin 3-O-glucoside, cyanidin 3-O-galactoside, cyanidin 3-O-arabinoside, peonidin 3-O-glucoside, peonidin 3-O-galactoside, peonidin 3-O-arabinoside	β-carotene, lutein
Custard apple	*Annona squamosa L.*	Catechin, epicatechin, epicatechin 3-O-gallate, procyanidin dimers (B1, B2, B3, B4, B5, B7), procyanidin trimers (C1, EEC)	-
Fig	*Ficus carica L.*	Quercetin, catechin, epicatechin, procyanidin dimer B3	-
Fruta-pão	*Artocarpus altilis*	Gallic acid, protocatechuic acid, caffeic acid, 5-caffeoylquinic acid, ferulic acid, p-coumaric acid, myricetin, apigenin	-
Grape (black)	*Vitis vinifera L.*	Caffeoyl tartatic acid, p-coumaroyl tartaric acid, quercetin, quercetin 3-O-galactoside, quercetin 3-O-glucuronide, myricetin, catechin, epicatechim epicatechin 3-O-gallate,	β-carotene, lutein, neoxanthin, violaxanthin, zeaxanthin

		epigallocatechin, cyanidin 3-O-glucoside, cyanidin 3-O-(6-p-coumaroyl-glucoside), delphinidin 3-O-glucoside, delphinidin 3-O-(6-acetylglucoside), malvidin 3-O-glucoside, malvidin 3-O-(6-acetyl-glucoside), malvidin 3-O-(6-p-coumaroyl-glucoside), peonidin 3-O-glucoside, peonidin 3-O-(6-p-coumaroyl-glucoside), petunidin 3-O-glucoside, petunidin 3-O-(6-p-coumaroyl-glucoside), resveratrol, resveratrol 3-O-glucoside, piceatannol, lariciresinol, matairesinol, secoisolariciresinol	
Grape (green)	*Vitis vinifera L.*	Caffeoyl tartaric acid, p-coumaroyl tartaric acid, quercetin, quercetin 3-O-glucuronide, quercetin 3-O-rutinoside, myricetin, kaempferol 3-O-glucoside, kaempferol 3-O-galactoside, isorhamnetin 3-O-glucoside, catechin, gallocatechin, epicatechin, epicatechin 3-O-gallate, epigallocatechin, procyanidin dimers (B1, B2, B3, B4), procyanidin trimer C1, procyanidin trimer EEC, prodelphinidin dimer B3, lariciresinol, matairesinol, pinoresinol, secoisolariciresinol	-
Guava	*Psidium guajava L.*	Gallic acid, vanillic acid, 5-caffeoylquinic acid, ferulic acid, p-coumaric acid, quercetin, kaempferol, catechin, epicatechin, naringenin, vanillin	β-carotene, lycopene
Jaboticaba	*Myrciaria jaboticaba Berg*	Gallic acid, ellagic acid, quercetin, cyanidin 3-O-glucoside, delphinidin 3-O-glucoside	-
Jackfruit	*Artocarpus heterophyllus*	-	α-carotene, β-carotene, β-cryptoxanthin, lutein, violaxanthin, zeaxanthin
Jambo	*Eugenia malaccensis L.*	Quercetin, catechin, cyanidin	-
Jenipapo	*Genipa americana L.*	Gallic acid, protocatechuic acid, p-coumaric acid, myricetin, apigenin	-
Kiwi	*Actinidia chinensis Planch*	Luteolin, catechin 3-O-gallate, epicatechin, epigallocatechin 3-O-gallate, procyanidin dimer B2, procyanidin trimer C1, lariciresinol, matairesinol, medioresinol, pinoresinol, secoisolariciresinol, syringaresinol	β-cryptoxanthin, lutein, zeaxanthin
Lemon	*Citrus limon*	Quercetin, luteolin, naringenin, hesperetin, eriodictyol, secoisolariciresinol	β-cryptoxanthin
Lychee	*Litchi chinensis Sonn.*	Gallic acid, caffeic acid, 5-caffeoylquinic acid, quercetin 3-O-rutinoside, catechin, epicatechin	-
Macaúba	*Acrocomia aculeata*	-	β-carotene
Mangaba	*Hancornia speciosa Gomes*	Gallic acid, protocatechuic acid, vanillic acid, 5-caffeoylquinic acid, p-coumaric acid, myricetin, kaempferol, apigenin, luteolin, isorhoifolin, cyanidin, pelargonidin, delphinidin	β-carotene, β-cryptoxanthin
Mango	*Mangifera indica L.*	Catechin, matairesinol, secoisolariciresinol	β-carotene, β-cryptoxanthin, neoxanthin, violaxanthin, zeaxanthin

Mari	*Poraqueiba sericea Tulasne*	Gallic acid, caffeic acid, ferulic acid, p-coumaric acid, quercetin, myricetin, apigenin, naringenin	-
Murici	*Byrsonima crassifolia*	Gallic acid, syringic acid, caffeic acid, ferulic acid, p-coumaric acid, quercetin, apigenin, luteolin	β-carotene, lutein, zeaxanthin
Nectarine	*Prunus persica var. nucipersica*	Quercetin, kaempferol	β-carotene, β-cryptoxanthin, lutein, violaxanthin, zeaxanthin
Orange	*Citrus sinensis L. Osbeck*	Quercetin, kaempferol, naringenin, hesperetin, lariciresinol, matairesinol, medioresinol, pinoresinol, secoisolariciresinol, syringaresinol	β-cryptoxanthin, lutein, zeaxanthin
Papaya	*Carica papaya L.*	4-Hydroxybenzoic acid, protocatechuic acid, caffeic acid, 5-caffeoylquinic acid, ferulic acid, p-coumaric acid, myricetin, kaempferol 3-O-glucoside, apigenin, luteolin	β-carotene, β-cryptoxanthin, lycopene
Passionfruit	*Passiflora edulis L.*	Secoisolariciresinol	β-carotene, β-cryptoxanthin
Peach	*Prunus persica L.*	3-Caffeoylquinic acid, 5-caffeoylquinic acid, 3-feruloylquinic acid, 3,p-coumaroylquinic acid, lariciresinol, matairesinol, pinoresinol, secoisolariciresinol	β-carotene, β-cryptoxanthin, lycopene, lutein, violaxanthin
Pear	*Pyrus communis*	5-Caffeoylquinic acid, gentisic acid, quercetin, quercetin 3-O-glucoside, quercetin 3-O-galactoside, quercetin 3-O-rutinoside, isorhamnetin 3-O-glucoside, catechin, epicatechin, lariciresinol, matairesinol, pinoresinol, secoisolariciresinol, arbutin	Lutein
Persimmon	*Diospyros kaki L.*	Catechin, gallocatechin, procyanidin dimers (B1, B3), procyanidin trimer EEC, prodelphinidin dimer B3	α-carotene, β-carotene, β-cryptoxanthin, lycopene, lutein, neoxanthin, violaxanthin, zeaxanthin
Physallis	*Physalis peruviana L.*	4-Hydroxybenzoic acid, benzoic acid, gallic acid, syringic acid, vanillic acid, cinnamic acid, o-coumaric acid, kaempferol, apigenin, naringenin, vanillin, catechol	α-carotene, β-carotene, β-cryptoxanthin, lutein, zeaxanthin
Pineapple	*Ananas comosus L. Merr.*	Lariciresinol, matairesinol, medioresinol, pinoresinol, secoisolariciresinol, syringaresinol	β-carotene, lutein
Pitanga	*Eugenia uniflora L.*	Quercetin, myricetin, kaempferol	β-carotene, β-cryptoxanthin, lycopene, lutein, violaxanthin
Plum	*Prunus domestica L.*	Protocatechuic acid, caffeic acid, 3-Caffeoylquinic acid, 4-caffeoylquinic acid, 5-caffeoylquinic acid, ferulic acid, 3-feruloylquinic acid, 5-feruloylquinic acid, p-coumaric acid, 3-p-coumaroylquinic acid, quercetin, quercetin 3-O-glucoside, quercetin 3-O-galactoside, quercetin 3-O-rutinoside, myricetin, kaempferol, catechin, epicatechin, cyanidin 3-O-glucoside, cyanidin 3-O-rutinoside, peonidin 3-O-glucoside, peonidin 3-O-rutinoside, procyanidin dimers (B1, B2, B3, B4, B5, B7), procyanidin trimer C1, procyanidin trimer EEC	β-carotene, β-cryptoxanthin, lutein
Soursoup	*Annona muricata*	Caffeic acid, 5-caffeoylquinic acid, ferulic acid, p-coumaric acid, apigenin, catechin, epicatechin	-
Star fruit	*Averrhoa carambola*	Catechin, epicatechin	β-carotene

Strawberry	*Fragaria L.*	4-Hydroxybenzoic acid 4-O-glucoside, galloyl glucose, ellagic acid, 5-caffeoylquinic acid, caffeoyl glucose, cinnamic acid, feruloyl glucose, p-coumaric acid, p-coumaric 4-O-glucoside, p-coumaroyl glucose, quercetin, kaempferol, epicatechin, epicatechin 3-O-gallate, epigallocatechin, gallocatechin, cyanidin, cyanidin 3-glucoside, cyanidin 3-O-(6-succinyl-glucoside), pelargonidin, pelargonidin 3-glucoside, pelargonidin 3-rutinoside, pelargonidin 3-O-(6-succinyl-glucoside), resveratrol, procyanidin dimers (B1, B2, B3, B4), procyanidin trimer EEC	β-carotene
Tangerine	*Citrus tangerina*	Lariciresinol, matairesinol, pinoresinol, secoisolariciresinol	β-cryptoxanthin, lutein, zeaxanthin
Tucumã	*Astrocaryum aculeatum*	-	α-carotene, β-carotene, β-cryptoxanthin, lutein, neoxanthin, violaxanthin, zeaxanthin
Uxi	*Endopleura uchi*	-	β-carotene
Watermelon	*Citrullus lanatus vulgaris*	Luteolin, lariciresinol, medioresinol, pinoresinol, secoisolariciresinol, syringaresinol	β-carotene, lycopene
**Vegetables**			
Arugula	*Eruca sativa Mill.*	Quercetin, kaempferol	β-carotene, lutein, neoxanthin, violaxanthin, zeaxanthin
Asparagus	*Asparagus officinalis*	Quercetin, kaempferol, isorhamnetin	β-carotene, lutein
Beetroot	*Beta vulgaris var. Rubra L.*	Gentisic acid, ferulic acid, quercetin, luteolin, secoisolariciresinol	β-carotene, lutein
Broccoli	*Brassica oleracea var. italica*	3-caffeoylquinic acid, 5-caffeoylquinic acid, 1-sinapoyl-2-feruloylgentiobiose, 1-sinapoyl-2,2-diferuloylgentiobiose, 1,2-disinapoylgentiobiose, 1,2-diferuloylgentiobiose, 1,2,2-trisinapoylgentiobiose, 1,2,2-triferuloylgentiobiose, 1,2-disinapoyl-2-feruloylgentiobiose, quercetin, quercetin 3-O-glucoside, quercetin 3-O-sophoroside, kaempferol, kaempferol 3-O-glucoside, kaempferol 3-O-sophoroside, kaempferol 3,7-O-diglucoside, catechin, lariciresinol, matairesinol, pinoresinol, secoisolariciresinol	β-carotene, lutein, neoxanthin, violaxanthin
Cabbage (green)	*Brassica oleracea var. capitata L.*	Quercetin, kaempferol, lariciresinol, matairesinol, pinoresinol, secoisolariciresinol	β-carotene, lutein
Cabbage (green)	*Brassica oleracea var. capitata L.*	Quercetin, apigenin, luteolin	-
Carrot	*Daucus carota sp. Sativus*	4-Hydroxybenzoic acid, protocatechuic acid, syringic acid, vanillic acid, caffeic acid, 3-caffeoylquinic acid, 4-caffeoylquinic acid, 5-caffeoylquinic acid, 3,4-dicaffeoylquinic acid, 3,5-dicaffeoylquinic acid, ferulic acid, 3-feruloylquinic acid, 4-feruloylquinic acid, 5-feruloylquinic acid, 3,4-diferuloylquinic acid, 3,5-diferuloylquinic acid, p-coumaric acid, 3-p-coumaroylquinic acid, 5-p-	α-carotene, β-carotene

		coumaroylquinic acid, quercetin, myricetin, kaempferol, luteolin, lariciresinol, matairesinol, pinoresinol, secoisolariciresinol	
Caruru	*Amaranthus viridis L.*	-	α-carotene, β-carotene, lutein, neoxanthin, violaxanthin
Cassava	*Manihot esculenta*	-	β-carotene
Cauliflower	*Brassica oleracea var. viridis L.*	Gallic acid, protocatechuic acid, syringic acid, caffeic acid, 5-caffeoylquinic acid, ferulic acid, sinapic acid	β-carotene, lutein
Celery	*Apium graveolens L.*	Quercetin, kaempferol, apigenin, luteolin, secoisolariciresinol, bergapten, isopimpinellin, psoralen, xanthotoxin	β-carotene
Chard	*Beta vulgaris var. flavescens*	4-Hydroxybenzoic acid, gallic acid, protocatechuic acid, syringic acid, vanillic acid, caffeic acid, 5-caffeoylquinic acid, ferulic acid, p-coumaric acid, quercetin, myricetin, kaempferol, secoisolariciresinol	α-carotene, β-carotene, lutein, violaxanthin
Chicory	*Cichorium intybus L.*	Quercetin, kaempferol, apigenin, luteolin	β-carotene, lutein, neoxanthin, violaxanthin
Chives	*Allium fistulosum L.*	Quercetin, kaempferol, isorhamnetin, luteolin	β-carotene, lutein
Chilli pepper	*Capsicum annuum*	Quercetin, luteolin	-
Coriander	*Coriandrum sativum L.*	Gallic acid, protocatechuic acid, caffeic acid, 5-caffeoylquinic acid, ferulic acid, quercetin	β-carotene, lutein, violaxanthin
Corn	*Zea mays L.*	4-Hydroxybenzoic acid, syringic acid, vanillic acid, caffeic acid, ferulic acid, p-coumaric acid, o-coumaric acid	β-carotene, β-cryptoxanthin, lutein, zeaxanthin
Cucumber	*Cucumis sativus L.*	Quercetin, kaempferol, apigenin, luteolin, lariciresinol, pinoresinol, secoisolariciresinol	β-carotene, lutein
Eggplant	*Solanum melongena L.*	4-Hydroxybenzoic acid, gallic acid, protocatechuic acid, caffeic acid, ferulic acid, p-coumaric acid, lariciresinol, medioresinol, pinoresinol, secoisolariciresinol, syringaresinol	β-carotene, lutein
Endive	*Cichorium endivia L.*	Kaempferol 3-O-glucoside, kaempferol 3-O-glucuronide, kaempferol 3-O-rhamnoside, kaempferol 3-O-(6-malonyl-glucoside)	β-carotene, lutein, neoxanthin, violaxanthin
Garlic	*Allium sativum L.*	Quercetin, myricetin, kaempferol, lariciresinol, matairesinol, pinoresinol, secoisolariciresinol	-
Ginger	*Zingiber P. Mill*	Secoisolariciresinol	-
Jalapeno pepper	*Capsicum annuum*	Quercetin, luteolin	α-carotene, β-carotene, lutein
Kale	*Brassica oleracea var. viridis L.*	Quercetin, kaempferol, isorhamnetin	β-carotene, lutein, neoxanthin, violaxanthin
Leek	*Allium ampeloprasum*	Quercetin, myricetin, kaempferol	β-carotene, lutein
Lettuce (green)	*Lactuca satia L.*	5-Caffeoylquinic acid, quercetin, quercetin 3-O-rutinoside, quercetin 3-O-rhamnoside, quercetin 3-O-glucuronide, quercertin 3-O-glucoside, quercetin 3-O-galactoside,	β-carotene, lutein, neoxanthin, violaxanthin

		quercetin 3-O-(6-malonyl-glucoside), quercetin 3-O-(6-malonyl-glucoside) 7-O-glucoside, myricetin, kaempferol, apigenin, luteolin, luteolin 7-O-glucuronide, lariciresinol, matairesinol, pinoresinol, secoisolariciresinol	
Lettuce (red)	*Lactuca satia L.*	Quercetin, quercetin 3-O-glucoside, quercetin 3-O-galactoside, quercetin 3-O-rhamnoside, quercetin 3-O-glucuronide, quercetin 3-O-(6-malonyl-glucoside), quercetin 3-O-(6-malonyl-glucoside) 7-O-glucoside, luteolin 7-O-glucuronide, cyanidin 3-O-glucoside, cyanidin 3-O-(6-malonyl-glucoside)	β-carotene, lutein
Mustard	*Brassica juncea var. rugosa*	Quercetin, kaempferol, isorhamnetin	α-carotene, β-carotene
Okra	*Hibiscus esculentus*		β-carotene
Onion (red)	*Allium cepa L. var. cepa*	Protocatechuic acid, quercetin, quercetin 3-O-glucoside, quercetin 3-O-rutinoside, quercetin 4-O-glucoside, quercetin 3,4-O-diglucoside, quercetin 7,4-O-diglucoside, myricetin, kaempferol, isorhamnetin, isorhamnetin 4-O-glucoside, apigenin, luteolin, cyanidin 3-O-(6-malonyl-glucoside), cyanidin 3-O-(6-malonyl-3-glucosyl-glucoside), delphinidin 3-O-glucosyl-glucoside	-
Onion (white)	*Allium cepa L. var. cepa*	Quercetin, quercetin 4-O-glucoside, quercetin 3,4-O-diglucoside	β-carotene, lutein
Ora-pro-nóbis	*Pereskia aculeata*	-	α-carotene, β-carotene
Parsley	*Petroselinum crispum Mill.*	Bergapten, isopimpinellin, psoralen, xanthotoxin	β-carotene, lutein, neoxanthin, violaxanthin
Pequi	*Caryocar villosium*	-	α-carotene, β-carotene, β-cryptoxanthin
Potato	*Solanum tuberosum L.*	Caffeic acid, 5-caffeoylquinic acid, quercetin, myricetin, kaempferol, apigenin, lariciresinol, matairesinol, secoisolariciresinol	β-carotene, lutein, zeaxanthin
Pumpkin	*Curcubita moschata*	Luteolin, lariciresinol, matairesinol, pinoresinol, secoisolariresinol	α-carotene, β-carotene, lutein, neoxanthin
Red pepper	*Capsicum annuum L. var. annuum*	-	β-carotene, lutein
Rice refined	*Oryza sativa L.*	4-Hydroxybenzoic acid, syringic acid, vanillic acid, ferulic acid, p-coumaric acid	-
Rice whole grain	*Oryza sativa L.*	4-Hydroxybenzoic acid, syringic acid, vanillic acid, caffeic acid, ferulic acid, p-coumaric acid	-
Scarlet eggplant	*Solanum gilo*	-	β-carotene
Serralha	*Sonchus oleraceus*	-	β-carotene, lutein, neoxanthin, violaxanthin
Spinach	*Spinacia oleracea L.*	Quercetin, kaempferol, 5,3,4-trihydroxy-3-methoxy-6:7-methylenedioxyflavone 4-O-glucuronide, 5,4-dihydroxy-3,3-dimethoxy-6:7-methylenedioxyflavone 4-O-	β-carotene, lutein, neoxanthin, violaxanthin

		glucuronide, jaceidin 4-O-glucuronide, patuletin 3-O-(2-feruloylglucosyl)(1->6)-[apiosyl(1->2)]-glucoside, patuletin 3-O-glucosyl-(1->6)-[apiosyl(1->2)]-glucoside, spinacetin 3-O-(2-feruloylglucosyl)(1->6)-[apiosyl(1->2)]-glucoside, spinacetin 3-O-(2-p-coumaroylglucosyl)(1->6)-[apiosyl(1->2)]-glucoside	
Sweet pepper (green)	*Capsicum annuum L.*	Feruloyl glucose, p-coumaroyl glucose, quercetin, quercetin 3-O-rhamnoside, luteolin, luteolin 7-O-(2-apiosyl-6-malonyl)-glucoside, lariciresinol, medioresinol, pinoresinol, secoisolariciresinol, syringaresinol	β-carotene, lutein, violaxanthin
Sweet pepper (red)	*Capsicum annuum L.*	Feruloyl glucose, p-coumaroyl glucose, quercetin, quercetin 3-O-rhamnoside, luteolin, luteolin 7-O-(2-apiosyl-6-malonyl)-glucoside, lariciresinol, pinoresinol, secoisolariciresinol	β-carotene, lutein, violaxanthin
Sweet pepper (yellow)	*Capsicum annuum L.*	Quercetin, luteolin, lariciresinol, pinoresinol, secoisolariciresinol	β-carotene, lutein, violaxanthin
Sweet potato	*Ipomoea batatas L.*	Lariciresinol, matairesinol, pinoresinol, secoisolariciresinol	β-carotene
Taioba	*Xanthosoma sagittifolium L.*	-	α-carotene, β-carotene, lutein, neoxanthin, violaxanthin
Tomato	*Lycopersicon esculentum Mill.*	Caffeic acid, 4-caffeoylquinic acid, 5-caffeoylquinic acid, ferulic acid, p-coumaric acid, quercetin, naringenin 7-O-glucoside	β-carotene, lycopene, lutein
Vinagreira	*Hibiscus sabdariffa L.*	-	β-carotene
Watercress	*Nasturtium officinale*	Protocatechuic acid, gentisic acid, ferulic acid, p-coumaric acid, quercetin, kaempferol, lariciresinol, pinoresinol, secoisolariciresinol	β-carotene, lutein, neoxanthin, violaxanthin
Yam	*Dioscorea trifida*	Quercetin	-
Zucchini	*Cucurbita pepo L.*	Gallic acid, caffeic acid, 5-caffeoylquinic acid, ferulic acid, p-coumaric acid, quercetin 3-O-rutinoside, kaempferol 3-O-glucoside	β-carotene, lutein
**Beans and derivates**			
Bean (black)	*Phaseolus vulgaris L.*	Caffeic acid, ferulic acid, p-coumaric acid, sinapic acid, quercetin, kaempferol, kaempferol 3-O-glucoside, kaempferol 3-O-acetyl-glucoside, daidzein, genistein, cyanidin, cyanidin 3-O-glucoside, cyanidin 3,5-O-diglucoside, delphinidin 3-O-glucoside, delphinidin 3-O-feruloyl-glucoside, malvidin 3-O-diglucoside, pelargonidin, pelargonidin 3-O-glucoside, pelargonidin 3,5-O-diglucoside, peonidin, petunidin 3-O-glucoside	-
Bean (carioca)	*Phaseolus vulgaris L.*	Ferulic acid, quercetin, kaempferol	-
Bean (white)	*Phaseolus vulgaris L.*	Ferulic acid, p-coumaric acid, sinapic acid, quercetin, kaempferol, kaempferol 3-O-acetyl-glucoside, kaempferol 3-O-glucoside, kaempferol 3-O-xylosyl-glucoside, catechin, epicatechin, procyanidin dimer B3, daidzein, genistein, matairesinol, secoisolariciresinol	-

Lentil	*Lens culinaris*	4-Hydroxybenzoic acid, protocatechuic acid, ferulic acid, p-coumaric acid, p-coumaroyl glycolic acid, p-coumaroyl malic acid, quercetin 3-O-rutinoside, myricetin 3-O-rhamnoside, apigenin 7-O-glucoside, apigenin 7-O-apiosyl-glucoside, luteolin, luteolin 7-O-glucoside, catechin, catechin 3-O-glucose, epicatechin, gallocatechin, resveratrol 3-O-glucoside, procyanidin dimers (B2, B3), prodelphinidin dimer B3,	-
Pea	*Pisum sativum L.*	Catechin, epicatechin	β-carotene, lutein
Soy (bean)	*Glycine Max L.*	Daidzein, daidzin, genistein, genistin, glycitein, glycitin, formononetin, 6-O-acetyldaidzin, 6-O-acetylgenistin, 6-O-acetylglycitin, 6-O-malonyldaidzin, 6-O-malonylgenistin, 6-O-malonylglycitin, lariciresinol, matairesinol, pinoresinol, secoisolariciresinol, coumestrol	-
Soy paste (miso)	*Glycine Max L.*	Daidzein, daidzin, genistein, genistin, glycitein, glycitin, 6-O-acetyldaidzin, 6-O-acetylgenistin, 6-O-acetylglycitin, 6-O-malonyldaidzin, 6-O-malonylgenistin, 6-O-malonylglycitin	-
Soy sauce	*Glycine Max L.*	Daidzein, genistein, genistin, glycitein, 6-O-acetylglycitin, formononetin, matairesinol, pinoresinol, secoisolariciresinol, coumestrol	-
Tofu	*Glycine Max L.*	Daidzein, daidzin, genistein, genistin, glycitein, glycitin, formononetin, 6-O-acetyldaidzin, 6-O-acetylgenistin, 6-O-acetylglycitin, 6-O-malonyldaidzin, 6-O-malonylgenistin, 6-O-malonylglycitin, lariciresinol, matairesinol, medioresinol, pinoresinol, secoisolariciresinol, syringaresinol, coumestrol	-
**Others**			
Almond	*Prunus dulcis*	4-Hydroxybenzoic acid, protocatechuic acid, vanillic acid, quercetin, quercetin 3-O-glucoside, quercetin 3-O-rutinoside, quercetin 3-O-galactoside, kaempferol, kaempferol 3-O-glucoside, kaempferol 3-O-rutinoside, kaempferol 3-O-galactoside, isorhamnetin, isorhamnetin 3-O-galactoside, 6,8-dihydroxykaempferol, naringenin, naringenin 7-O-glucoside, eriodictyol, catechin, gallocatechin 3-O-gallate, epicatechin, epigallocatechin, lariciresinol, matairesinol, pinoresinol, secoisolariciresinol	-
Basil	*Ocimum basilicum L.*	Vanillic acid, rosmarinic acid	β-carotene, lutein
Beer		2-Hydroxybenzoic acid, 3-hydroxybenzoic acid, 4-hydroxybenzoic acid, 2,6-dihydroxybenzoic acid, 3,5-dihydroxybenzoic acid, gallic acid, gallic acid 3-O-gallate, gentisic acid, protocatechuic acid, syringic acid, vanillic acid, caffeic acid, 4-caffeoylquinic acid, 5-caffeoylquinic acid, ferulic acid, m-coumaric acid, p-coumaric acid, o-coumaric acid, sinapic acid, 4-hydroxyphenylacetic acid, homovanillic acid, quercetin, quercetin 3-O-rutinoside, quercetin 3-O-arabinoside, myricetin, 3,7-dimethylquercetin, apigenin, naringin, isoxanthohumol, 6-geranylnaringenin, 6-prenylnaringenin, 6-prenylnaringenin, biochanin A, catechin, epicatechin, xanthohumol, procyanidin dimer B3, procyanidin trimer C2, prodelphinidin dimer B3,	-

		prodelphinidin trimers (C-GC-C, GC-C-C, GC-GC-C), matairesinol, secoisolariciresinol, 4-vinylguaiacol, 3-methylcatechol, 4-ethylcatechol, 4-vinylphenol, vanillin, 2,3-dihydroxy-1-guaiacylpropanone, 4-hydroxycoumarin, esculin, umbelliferone, tyrosol, catechol, pyrogallol	
Brazil nut	*Bertholletia excelsa*	Matairesinol, secoisolariciresinol	-
Cashew nut	*Anacardium occidentale L.*	Catechin, epicatechin, epicatechin 3-O-gallate	β-carotene, lutein, zeaxanthin
Cassava flour	*Manihot esculenta*	-	β-carotene
Chestnut	*Castanea P. Mill*	Gallic acid, ellagic acid, catechin, gallocatechin, procyanidin dimer B3, procyanidin trimer C1, lariciresinol, matairesinol, pinoresinol, secoisolariciresinol	-
Chocolate (milk)		Catechin, epicatechin	-
Chocolate (dark)		Ferulic acid, quercetin, catechin, epicatechin, epicatechin-(2a-7)(4a-8)-epicatechin 3-O-galactoside, procyanidin dimer B2, procyanidin trimer C1, cinnamtannin A2, resveratrol, resveratrol 3-O-glucoside	-
Cinnamon		Cinnamic acid, coumarin	-
Cloves	*Syzygium aromaticum*	Gallic acid, protocatechuic acid, syringic acid, p-coumaric acid, quercetin, kaempferol, eugenol, acetyl eugenol	-
Cocoa powder	*Theobroma cacao*	Benzoic acid, protocatechuic acid, syringic acid, vanillic acid, caffeoyl aspartic acid, catechin, epicatechin, epicatechin-(2a-7)(4a-8)-epicatechin 3-O-galactoside, procyanidin dimers (B1, B2), procyanidin trimer C1, cinnamtannin A2, secoisolariciresinol, 3-methylcatechol, 4-ethylcatechol, 4-methylcatechol, vanillin, mellein, catechol, phenol, pyrogallol	-
Coffee	*Coffea arabica L.*	4-hydroxybenzoic acid, vanillic acid, caffeic acid, 3-caffeoylquinic acid, 4-caffeoylquinic acid, 5-caffeoylquinic acid, 3,4-dicaffeoylquinic acid, 3,5-dicaffeoylquinic acid, 4,5-dicaffeoylquinic acid, feruic acid, 3-feryloylquinic acid, 4-feruloylquinic acid, cinnamic acid, p-coumaric acid, lariciresinol, matairesinol, pinoresinol, secoisolariciresinol, 4-ethylguaiacol, 4-vinylguaiacol, 3-methylcatechol, 4-methylcatechol, 4-ethylcatechol, guaiacol, catechol, phenol, pyrogallol	-
Corn flour	*Zea mays L.*	2-Hydroxybenzoic acid, 4-hydroxybenzoic acid, protocatechuic acid, syringic acid, vanillic acid, caffeic acid, ferulic acid, p-coumaric acid, 5,5-dehydrodiferulic acid, 5,8-benzofuran dehydrodiferulic acid, 5,8-dehydrodiferulic acid, 8-O-4-dehydrodiferulic acid, 4-hydroxyphenylacetic acid	β-carotene, β-cryptoxanthin, lutein
Dende oil	*Elaeis guineensis*	-	α-carotene, β-carotene, lycopene
Egg yolk			Lutein, zeaxanthin
Hazelnut	*Corylus L.*	Catechin, epicatechin, epigallocatechin, epigallocatechin 3-O-gallate, gallocatechin 3-O-gallate, lariciresinol, matairesinol, pinoresinol, secoisalariciresinol	-

Honey bee		Protocatechuic acid, vanillic acid, caffeic acid, 5-caffeoylquinic acid, ferulic acid, rosmarinic acid, p-coumaric acid, quercetin, myricetin, kaempferol, isorhamnetin, apigenin, luteolin, naringenin, eriodictyol, syringaldehyde	-
Lucuri	*Syagrus coronata (Martius) Beccari*	Quercetin, quercetin 3-O-glucoside, quercetin 3-O-rutinoside, quercetin 3-O-rhamnoside, myricetin, catechin, epicatechin, procyanidin dimers (B1, B2)	β-carotene, β-cryptoxanthin, lycopene
Oat flakes	*Avena sativa L.*	4-hydroxybenzoic acid, syringic acid, vanillic acid, caffeic acid, ferulic acid, sinapic acid, p-coumaric, avenanthramides (A, B, C), 4-hydroxybenzaldehyde	-
Oat flour	*Avena sativa L.*	4-hydroxybenzoic acid, protocatechuic acid, syringic acid, vanillic acid, caffeic acid, p-coumaric acid, ferulic acid, sinapic acid, avenanthramide B, 4-hydroxyphenylacetic acid	-
Olive (black)	*Olea europaea L.*	4-Hydroxybenzoic acid, 2,4-dihydroxybenzoic acid, 2,6-dihydroxybenzoic acid, gallic acid, protocatechuic acid, syringic acid, vanillic acid, caffeic acid, 5-caffeoylquinic acid, cinnamic acid, ferulic acid, m-coumaric acid, o-coumaric acid, p-coumaric acid, sinapic acid, verbascoside, 3,4-dihydroxyphenylacetic acid, 4-hydroxyphenylacetic acid, homovanillic acid, methoxyphenylacetic acid, homoveratric acid, dihydro-p-coumaric acid, dihydrocaffeic acid, quercetin 3-O-rutinoside, quercetin 3-O-rhamnoside, apigenin, isorhoifolin, luteolin, luteolin 6-C-glucoside, luteolin 7-O-glucoside, cyanidin 3-O-glucoside, cyanidin 3-O-rutinoside, vanillin, 3,4-DHPEA-EA, demethyloleuropein, hydroxytyrosol, oleuropein, tyrosol, lariciresinol, matairesinol, pinoresinol, secoisolariciresinol	-
Olive (green)	*Olea europaea L.*	4-Hydroxybenzoic acid, protocatechuic acid, syringic acid, vanillic acid, caffeic acid, cinnamic acid, ferulic acid, m-coumaric acid, o-coumaric acid, p-coumaric acid, sinapic acid, verbascoside, 3,4-dihydroxyphenylacetic acid, 4-hydroxyphenylacetic acid, homovanillic acid, dihydro-p-coumaric acid, dihydrocaffeic acid, luteolin, tyrosol, hydroxytyrosol, 3,4-DHPEA-EA, demethyloleuropein, oleuropein, oleoside 11-methylester, 3,4-dihydroxyphenylglycol, lariciresinol, matairesinol, pinoresinol, secoisolariciresinol	-
Olive oil virgin	*Olea europaea L.*	2,4-Dihydroxybenzoic acid, 2,6-dihydroxybenzoic acid, 4-hydroxybenzoic acid, protocatechuic acid, syringic acid, vanillic acid, caffeic acid, 5-caffeoylquinic acid, ferulic acid, m-coumaric acid, p-coumaric acid, 4-hydroxyphenylacetic acid, 3,4-dihydroxyphenylacetic acid, homovanillic acid, homoveratric acid, apigenin, luteolin, 1-acetoxypinoresinol, pinoresinol, vanillin, tyrosol, hydroxytyrosol, 3,4-DHPEA-AC, p- DHPEA-AC, 3,4-DHPEA-EA, p-DHPEA-EA, 3,4-DPHEA-EDA, p-DPHEA-EDA, oleuropein, ligstroside, 3,4-dihydroxyhenylglycol	-
Peanut	*Arachis hypogaea L.*	Genistein, daidzein, biochanin A, resveratrol, lariciresinol, matairesinol, secoisolariciresinol	-

Pecan nut	*Caryna illinoinensis*	Catechin, gallocatechin 3-O-gallate, epicatechin, epigallocatechin, epigallocatechin 3-O-gallate	-
Pine nut	*Araucaria angustifólia*	Gallic acid, quercetin, catechin	-
Pistachio	*Pistacia vera L.*	Catechin, gallocatechin 3-O-gallate, epicatechin, epigallocatechin	-
Saffron	*Crocus sativus L.*	Kaempferol 3,7,4-O-triglucoside, kaempferol 3-O-sophoroside, kaempferol 3-O-sophoroside 7-O- glucoside	β-carotene
Sesame seed	*Sesamum indicum L.*	Isohydroxymatairesinol, sesamin, sesaminol, sesamolin, 7-hydroxymatairesinol, 7-hydroxysecoisolariciresinol, 7-oxomatairesinol, arctigenin, conidendrin, dimethylmatairesinol, lariciresinol, matairesinol, medioresinol, secoisolariciresinol, syringaresinol, todolactol A, 4-vinylguaiacol	-
Tea (black)	*Camellia sinensis L.*	Quercetin, myricetin, kaempferol, epicatechin, epigallocatechin, theaflavin, theaflavin 3-O-gallate, theaflavin 3,3-O-digallate	-
Tea (green)	*Camellia sinensis L.*	Quercetin, myricetin, kaempferol, catechin, epicatechin, epigallocatechin	-
Tea (mate)	*Ilex paraguariensis*	Quercetin, kaempferol	-
Walnut	*Juglans regia L.*	Ellagic acid	-
Wheat flour (refined)	*Triticum aestivum L.*	Syringic acid, vanillic acid, ferulic acid, apigenin 6,8-C-galactoside-C-arabinoside, apigenin 6,8-C-arabinoside-C-glucoside, 5-heneicosylresorcinol, 5-nonadecenylresorcinol, 5-nonadecylresorcinol, lariciresinol, matairesinol, pinoresinol, secoisolariciresinol	-
Wheat flour (whole grain)	*Triticum aestivum L.*	Caffeic acid, ferulic acid, p-coumaric acid, sinapic acid, apigenin 6,8-C-galactoside-C-arabinoside, apigenin 6,8-C-arabinoside-C-glucoside, lariciresinol, matairesinol, medioresinol, pinoresinol, secoisolariciresinol, syringaresinol, 5-heneicosenylresorcinol, 5-heneicosylresorcinol, 5-heptadecylresorcinol, 5-nonadecenylresorcinol, 5-nonadecylresorcinol, 5-pentacosenylresorcinol, 5-pentacosylresorcinol, 5-pentadecylresorcinol, 5-tricosenylresorcinol, 5-tricosylresorcinol	-
Wine (red)	*Vitis vinifera L.*	2,3-Dihydroxybenzoic acid, 2-hydroxybenzoic acid, 4-hydroxybenzoic acid, gallic acid, gallic acid ethyl ester, gentisic acid, protocatechuic acid, syringic acid, vanillic acid, 2,5-di-S-Glutathionyl caftaric acid, caffeic acid, caffeoyl tartaric acid, ferulic acid, o-Coumaric acid, p-Coumaric acid, p-Coumaroyl tartaric acid, sinapic acid, 4-hydroxyphenylacetic acid, isorhamnetin, isorhamnetin 3-O-glucoside, kaempferol, kaempferol 3-O-glucoside, myricetin, quercetin, quercetin 3-O-arabinoside, quercetin 3-O-glucoside, quercetin 3-O-rhamnoside, quercetin 3-O-rutinoside, catechin, gallocatechin, epicatechin, epicatechin 3-O-gallate, epigallocatechin, hesperetin, naringenin, naringin, dihydromyricetin 3-O-rhamnoside, dihydroquercetin 3-O-	-

		rhamnoside, procyanidin dimers (B1, B2, B3, B4, B7), procyanidin trimer C1, procyanidin trimer T2, prodelphinidin dimer B3, cyanidin 3-O-(6-acetyl-glucoside), cyanidin 3-O-glucoside, delphinidin 3-O-(6-acetyl-glucoside), delphinidin 3-O-(6-p-coumaroyl-glucoside), delphinidin 3-O-glucoside, malvidin 3-O-(6-acetyl-glucoside), malvidin 3-O-(6-caffeoyl-glucoside), malvidin 3-O-(6-p-coumaroyl-glucoside), malvidin 3-O-glucoside, peonidin 3-O-(6-acetyl-glucoside), peonidin 3-O-(6-p-coumaroyl-glucoside), peonidin 3-O-glucoside, petunidin 3-O-(6-acetyl-glucoside), petunidin 3-O-(6-p-coumaroyl-glucoside), petunidin 3-O-glucoside, pigment A, pinotin A, vitisin A, resveratrol, resveratrol 3-O-glucoside, d-Viniferin, e-Viniferin, pallidol, piceatannol, piceatannol 3-O-glucoside, protocatechuic aldehyde, syringaldehyde, hydroxytyrosol, tyrosol	
Wine (white)	*Vitis vinifera L.*	2-hydroxybenzoic acid, 4-hydroxybenzoic acid, gallic acid, gentisic acid, protocatechuic acid, syringic acid, vanillic acid, 5-caffeoylquinic acid, caffeic acid, caffeoyl tartaric acid, ferulic acid, o-coumaric acid, p-coumaric acid, sinapic acid, 4-hydroxyphenylacetic acid, naringin, 3,7-dimethylquercetin, kaempferol, quercetin, quercetin 3-O-arabinoside, quercetin 3-O-rutinoside, catechin, gallocatechin, epicatechin, epicatechin 3-O-gallate, malvidin 3-O-glucoside, dihydromyricetin 3-O-rhamnoside, dihydroquercetin 3-O-rhamnoside, procyanidin dimers (B1, B2, B3, B4), resveratrol, resveratrol 3-O-glucoside, e-viniferin, pallidol, piceatannol 3-O-glucoside, protocatechuic aldehyde, 4-hydroxycoumarin, hydroxytyrosol, tyrosol	-

-: content equal to zero or not described.
